# XXX/XY chimerism with urogenital malformations in a Japanese black calf

**DOI:** 10.1186/s13620-025-00301-7

**Published:** 2025-08-27

**Authors:** Chihiro Kanno, Makoto Sugiyama, Hiroshi Miura, Sayori Ozawa, Shogo Sato, Chiharu Kudo, Hiroaki Kawaguchi

**Affiliations:** 1 Kitasato University School of Veterinary Medicine, 35-1 Higashi-23bancho, Towada, Aomori 034-8628 Japan; 2 Hokkaido Obihiro Meat Inspection Center, Towada, Japan; 3 Kudo Animal Clinc, Mutsu, Japan

**Keywords:** Japanese black calf, Sex chromosome abnormality, Urogenital malformations, X-trisomy, Freemartin, Background

## Abstract

**Background:**

Sex chromosome abnormalities in cattle are rare, and manifestations of genital anomalies due to such abnormalities are even less frequently reported. Among these, XXX/XY chimerism is particularly uncommon. This report presents a Japanese black calf with complex urogenital malformations linked to XXX/XY chimerism, contributing valuable insights into bovine sex determination and reproductive development.

**Case presentation:**

A Japanese black calf of phenotypic indeterminate sex, born co-twin to a phenotypically normal male, presented with hypospadias-like features and ambiguous genitalia. Clinical examination revealed a scrotum-like structure without palpable testes or vulva. An hCG stimulation test indicated a lack of functional testicular tissue. Chromosomal analysis of leukocytes revealed the presence of two distinct cells with 60, XY and 61, XXX, revealing XXX/XY chimerism. The ratio of male to trisomic cells was 63:37 (95% confidence intervals; XY: 54–72%, XXX: 28–46%) in the affected calf. Necropsy revealed both male (testis, epididymis, ductus deferens) and female (uterus-like) reproductive structures, with uterus-like organs embedded within the perineal tissue. Histological and immunohistochemical analyses confirmed the presence of the uterine remnant and revealed Sertoli cell-only testicular tissue, indicating spermatogenic failure. PCR-based sex determination performed on multiple tissues revealed three distinct genotypic patterns, with evidence of tissue-specific variation in the distribution of the X and Y chromosomes. Some tissues lacked detectable Y-linked AMEL-Y, despite the presence of SRY, suggesting a complex chimeric constitution with potential deletion of the AMEL-Y region in some XY cell populations.

**Conclusions:**

This case highlights a rare instance of systemic XXX/XY chimerism associated with ambiguous genitalia and mixed internal reproductive structures, which is distinct from typical freemartinism or isolated X-trisomy syndromes. The differential chromosomal mosaicism across tissues likely influences the phenotypic outcome. These findings emphasize the complexity and plasticity of bovine sex differentiation, particularly in twin pregnancies, and underscore the importance of integrating clinical, cytogenetic, and molecular diagnostics to accurately identify and manage congenital reproductive anomalies in livestock.

## Background

Sex chromosome abnormalities generally do not result in malformations because one of the X chromosomes is genetically inactivated to compensate for the gene dosage [1]. X-trisomy is the most common sex chromosome abnormality in humans, and infertility occurs only when multiple X chromosomes are inadequately inactivated [2]. Various physical characteristics, such as tall stature, hypotonia, clinodactyly, and increased incidence of autoimmunity, are present in women with trisomy X [3]. In addition, reproductive disorders, including diminished ovarian reserve and accelerated loss of ovarian function, are observed in these cases, although these symptoms are not inevitable [4]. In cattle, on the other hand, the prevalence of sex chromosome aneuploidy is very low (XO: 0.0048, XXX: 0.0350, XXY: 0.0004%, respectively) [5], and a few cases of genital tract aneuploidy due to X-trisomy have been reported [6–10]. Infertility is caused abnormalities in internal sexual structures, such as ovarian hypoplasia, atrophic uterine body, and a lack of estrus [11].

The most common sex chromosome abnormalities in cattle are XX/XY mosaicism and freemartinism, which are found in approximately 90% of twins of different sexes [12]. Freemartin is defined as a female calf born co-twin to a male calf that exchanges blood components with its male sibling in utero, resulting in the suppressed development of the female reproductive tract and varying degrees of enhanced development of the male reproductive tract [13]. In freemartins, although the external genital tract is similar to that of normal females, ovaries show different degrees of testicularization, and some cases exhibit internal genitalia of both sexes. Freemartins have low economic value because of their infertility and low meat production productivity [14].

In this case, we examined a Japanese black calf with XXX/XY chimeras and urogenital malformation. This case had features that differed from the genital abnormalities observed in typical freemartins. Moreover, different gene expression patterns in different tissues were confirmed in the present case. The relationships between sex chromosome mosaics and anatomical characteristics are poorly understood in cattle, except for typical freemartinism. Japanese black cattle have high economic value because their meat is sold at a higher price than other meat breeds of foreign origin [15]. Therefore, we believe that the presentation of the clinical, anatomical, and molecular features of this case will be valuable from both academic and economic perspectives. Herein, we describe a case of XXX/XY mosaicism with urogenital malformations in a Japanese black calf.

## Case presentation

The subject of this report was a Japanese black calf of indeterminate sex, which was born as a co-twin with an externally normal-appearing male calf. The calf presented to the university hospital at one month of age (Day 0). Upon initial examination, the calf exhibited hypospadias-like abnormalities of the urinary tract. The penis, testis, and vulva were not palpable, although a scrotum-like structure was observed.

A human chorionic gonadotropin (hCG) stimulation test was performed to diagnose cryptorchidism. Three thousand units of hCG (Gestron, Kyoritsu Pharmaceutical, Tokyo, Japan) were administered intramuscularly. Blood samples were collected before and 24 h after hCG administration and then centrifuged immediately after collection to obtain plasma. The plasma was stored at −30 °C until it was used for testosterone measurement. Plasma testosterone levels were evaluated via competitive ELISA with slight modifications to a previous study [6]. A rabbit anti-testosterone antibody (KZ-HS-P14, Cosmobio, Tokyo, Japan) was used as the primary antibody, and a goat anti-rabbit IgG-HRP (4030–05, SouthernBiotech, Birmingham, Albania) was used as the secondary antibody. The assay was performed in a 96-well plastic plate precoated with antibodies.

Loop-mediated isothermal amplification (LAMP) was performed to investigate sex chromosomal chimerism in the present case and in its family. Blood samples were collected in EDTA tubes from the case, its sibling, and its dam. A Loopamp bovine embryo sexing kit (Eiken Chemical, Tokyo, Japan) was used according to the manufacturer’s instructions. Chromosome counting was performed via microscopic observation of metaphase chromosomes in leukocytes following a previously reported method [16]. Briefly, blood samples were collected into heparinized tubes from the case, its half-sibling, and the dam. The samples were cultured in 199 medium supplemented with 0.1 ml of PHA-M and 10% calf serum at 37 °C and 5% CO_2_ for 3 days, and 70 h after the start of culture, the colcemid (0.5 µg/ml; colchicine tablet 0.5 mg “Takata”, Takata pharmaceutical, Saitama, Japan) was added to maximize the number of leukocytes in metaphase. After culture, the blood samples were treated with 0.075 M KCl, and the leukocytes were spread onto glass slides. One hundred cells were analysed to count the number of chromosomes and determine the type of sex chromosome (XX, XY, or XXX). Finally, the population for each sex chromosome type and their 95% confidence intervals were calculated.

Although the malformed calf exhibited no health problems, it was euthanized and necropsied on day 10. At necropsy, systemic organs, including uterus-like structures, were fixed in 10% phosphate-buffered formalin and routinely processed into paraffin-embedded tissue sections with a thickness of 5 μm. The sections were stained with hematoxylin and eosin (HE). The uterus-like structure was stained with Masson’s trichrome (MT). Furthermore, immunohistochemical staining was performed on the uterus-like remnant using a monoclonal anti-alpha smooth muscle actin (α-SMA) antibody (1:50; Dako, Tokyo, Japan) with an EnVision kit (Dako). Similarly, immunostaining was conducted on the testis via a monoclonal anti-vimentin antibody (Applied undiluted; Dako) and an EnVision kit.

The liver, spleen, thyroid gland, urachus, testis, epididymis, deferent duct, kidney, ureter, urinary bladder, penis, and uterus-like structure were collected for polymerase chain reaction (PCR). PCR was performed to identify the X and Y chromosomes. Genomic DNA was extracted from each tissue sample via a standard protocol that included proteinase K digestion, followed by phenol-chloroform-isoamyl alcohol (PCI) extraction. PCR-based chromosomal sex determination was performed as previously described [17], targeting the bovine SRY and amelogenin (AMEL) loci. The primers used to amplify a 480 bp fragment of the SRY region were 5’-TCGTGAACGAAGACGAAAGGTGGC-3’ (forward) and 5’-GCACAAGAAAGTCCAGGCTCTAAGC-3’ (reverse). For the AMEL locus, primers were designed to amplify a 280 bp fragment from the X chromosome and a 217 bp fragment from the Y chromosome via the primers 5’-CAGCCAAACCT-CCCTCTGC-3’ (forward) and 5’-CCCGCTTGGTCTTGTCTGTTGC-3’ (reverse). PCR was performed via KOD-FX neo (Toyobo, Osaka, Japan) under the following conditions: 36 cycles at 94 °C for 10 s, 66 °C for 30 s, and 68 °C for 30 s.

## Results and discussions

In the hCG stimulation test, the pre- and post- testosterone levels were both below 0.1 ng/ml, suggesting that the patient did not have functional testes that could respond to luteinising hormone (LH) stimulation.

**Fig. 1:**
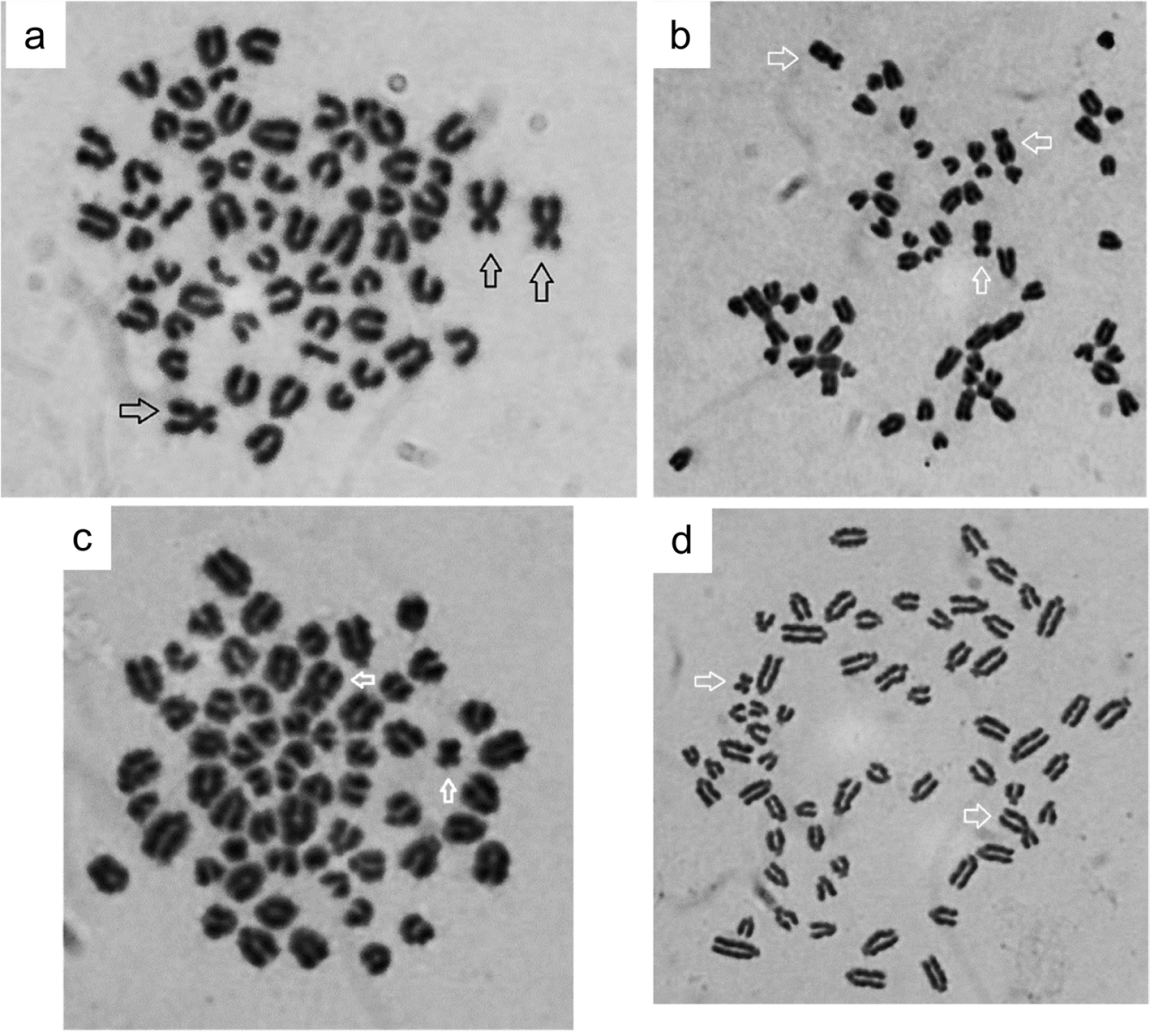
Leukocyte karyotypes of the affected bovine (Case) and its sibling. Representative karyotypes determined by leukocyte chromosome examination. **a** XXX and **b** XY karyotypes from the patient, indicating XY/XXX mosaicism. **c** XXX and **d** XY karyotypes from its sibling. Arrow: Sex chromosomes

The LAMP test indicated that the case and its half-sib had both X and Y chromosomes, whereas the dam only had X chromosomes. As shown in Fig. 1, microscopic observation of chromosomes in leukocytes revealed a mixture of X chromosome trisomy cells (61/XXX, Fig. 1a, c) and male cells (60/XY, Fig. 1b, d) in both twins. Notably, the ratios of male cells to trisomy 100 cells for the normal male sibling were 81% (60/XY) and 19% (61/XXX), whereas those for the malformed calf were 63% (60/XY) and 37% (61/XXX), respectively. In contrast, only female cells (60/XX) were observed in the mother cow. These results suggest that the somatic cells of the malformed calf had X chromosome trisomy and exhibited the (XXX/XY) chromosome type due to the exchange of blood components between twins when they were fetuses. These findings suggest a profound masculinization of an originally XXX female, driven by its XY co-twin. This scenario is distinct from classic freemartinism, which typically involves the incomplete transformation of an XX female. While this interpretation is the most parsimonious explanation for the calf's phenotype, the ultimate origin of the XXX cells remains unproven. The phenotypically normal male co-twin was also a chimera, and though its fertility was not assessed, potential impacts on the reproductive health of such bulls are a recognized concern [14]. This case highlights the complex outcomes of chimerism in cattle, potentially involving mechanisms beyond the classic XX/XY model.

**Fig. 2:**
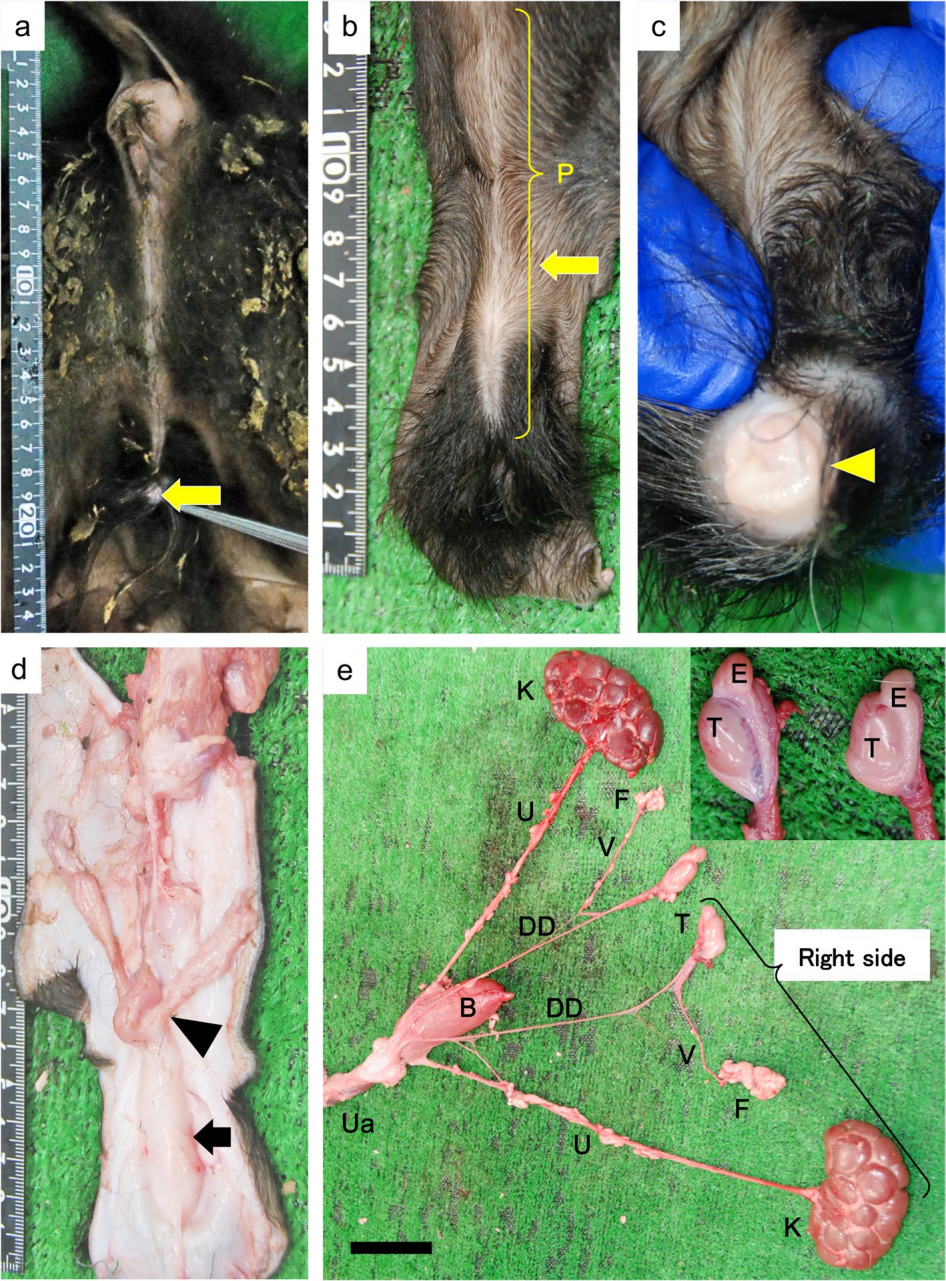
Gross findings of the urogenital tract. **a** Hypospadias (arrow) is observed approximately 12 cm below the anus. **b** Penis and orifices (arrows) are visible. **c** The tip of the penis (arrowhead) is visible at the opening. **d** Inside view of hypospadias (arrow): Uterus-like structures that are Y-shaped and mimic a bicornuate uterus (arrowhead) are continuous with the hypospadias. **e** Urogenital: the kidneys, ureters, bladder and urethra are located in the urinary system. Testes and deferent ducts are observed in the male genital tract. Fatty tissue mass areas are observed around the testes but do not include ovarian tissue. Bar = 5 cm. Insert: Testes and epididymides. B: bladder, DD: deferent duct, E: epididymis, F: fatty tissue mass, K: kidney, P: penis, T: testis, U: ureter, Ua: urethra, V: vessel

Necropsy of the malformed calf (Fig. 2) revealed a pseudolabia-like hypospadia structure in the area corresponding to the perineum (12 cm below the midline from just below the anus; Fig. 2a-c). Uterus-like structures were observed in the subcutaneous tissue at the hypospadias site. The uterus-like structures were Y shaped and mimicked a bicornuate uterus that was continuous with hypospadias (Fig. 2d). However, male reproductive organs (testes, epididymis, seminal vesicle gland, urethra, and penis, Fig. 2e) were also observed.

**Fig. 3:**
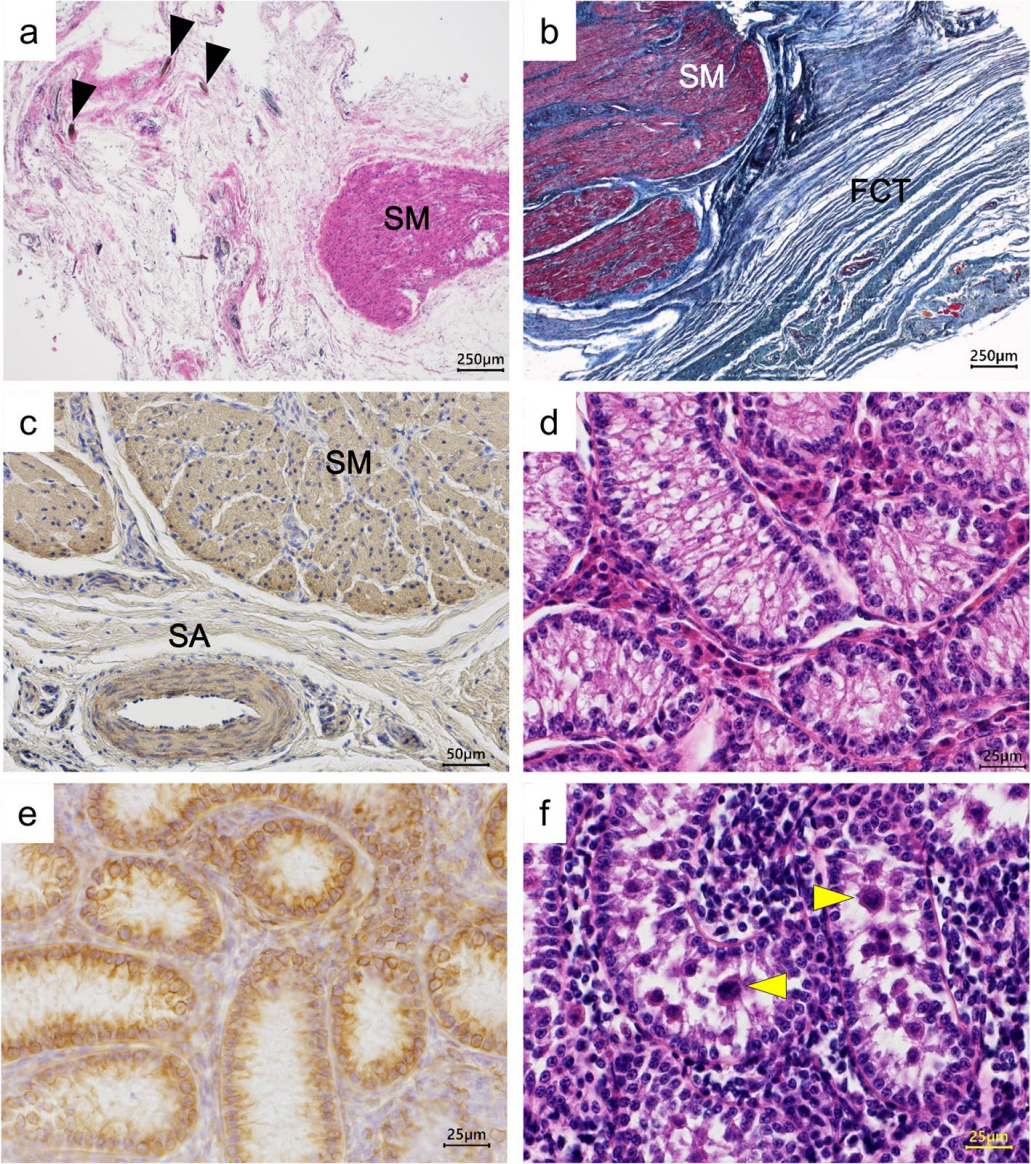
Histological examination of the uterus-like structure and testis. **a**-**c** Uterus-like structure: The structure consists of masses of smooth muscle, which are stained red with MT and are positive for α-SMA immunostaining **c**. Fibrous connective tissue, which stains blue with Masson’s trichrome staining (**b**), is observed around the structure, and hairs (arrowheads) are also observed in the fibrous connective tissue. **d**, **e** Testis: Only Sertoli cells that are positive for vimentin immunostaining are observed in the seminiferous tubules. **f** Testis in a healthy calf of a similar age: Sertoli cells and spermatogonia (arrowhead) are observed in the seminiferous tubules. **a**, **d**, **f**: HE staining, b: MT staining, c: α-SMA immunostaining, e: vimentin immunostaining. FCT: fibrous connective tissue, HE: hematoxylin and eosin, MT: Masson’s trichrome, SA: small artery, SM: smooth muscle, SMA: smooth muscle actin

Histological examination (Fig. 3) revealed that the uterus-like structures consisted of masses of smooth muscle, which were stained red with MT and immunohistochemically positive for α-SMA. Fibrous connective tissue, which was stained blue with MT, was observed around uterus-like structures (Fig. 3a-c). Hairs were also observed in the fibrous connective tissue, suggesting that they were derived from skin appendages of the labia. Therefore, the uterus-like structures were suggested to be remnants of the female reproductive organs. In the testes, the seminiferous tubules were composed only of Sertoli cells (Fig. 3d, e), and the spermatogonia found in healthy male calves of roughly the same age (Fig. 3f) were not observed, suggesting that calf malformation is a congenital abnormality of sperm agenesis.

**Fig. 4:**
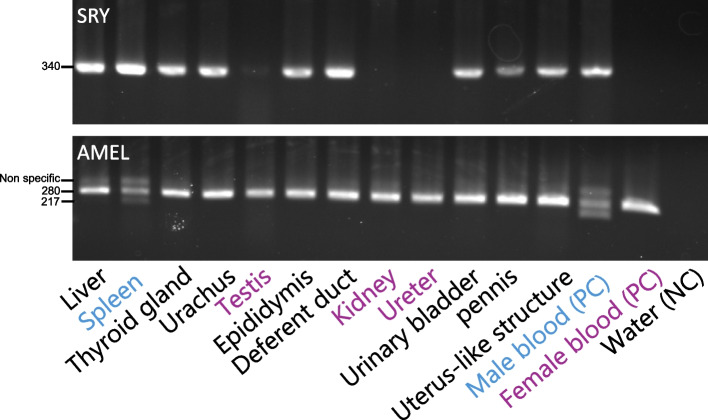
PCR analysis of Y and X chromosome-specific sequences in various tissues. The AMEL gene (a product of 217 bp on the Y chromosome and 280 bp on the X chromosome) and the SRY locus (340 bp) were detected via PCR in each tissue. Note that the 380 bp AMEL amplification product in males is a nonspecific sequence; PC, positive control; NC, negative control. Water was used as the template for the negative controls. The blue text indicates male sex gene amplification, the magenta text indicates female sex gene amplification features, and the black text indicates organs with single amplification of the SRY and AMEL genes

PCR analysis of the SRY and AMEL genes across various tissues revealed three different patterns (Fig. 4): (i) In the spleen, both SRY and AMEL fragments corresponding to X and Y chromosomes (AMEL-X and AMEL-Y) were detected, indicating a mixture of XXX and XY cell populations; (ii) In the kidney, testis and ureter, only the AMEL-X fragment was detected, with no SRY signal, suggesting a predominance of XXX cells and an XY contribution below the detection threshold; (iii) In the liver, SRY and AMEL-X were detected, but AMEL-Y was absent or undetectable (SRY +, AMEL-X +, AMEL-Y -). The third pattern presents an interpretive challenge and sporadic case. This might reflect the presence of an aberrant XY cell line carrying a Y chromosome with a deletion encompassing the AMEL-Y locus while retaining the SRY region, which coexists with XXX cells [18]. Overall, the variation in genetic patterns among tissues supports the presence of systemic chimerism. These relationships could be analysed in more detail using quantitative PCR.

The phenotype observed in the present case differed from both typical XX/XY freemartinism and previously reported XXX syndrome cases [5, 18]. This calf exhibited remnants of both Müllerian-derived (uterus-like structures) and Wolffian-derived structures (testis, epididymis, deferent duct), with particularly notable development of the testis and epididymis. This unique constellation of features likely results from the complex interactions between the coexisting XXX and XY cell lines. Furthermore, the dynamics of X-chromosome inactivation within the XXX cell population may have played a critical role in modulating the final phenotype, a factor that distinguishes this case from typical XX/XY freemartinism. Harikae et al. reported the formation of testes and seminiferous tubules lacking gametes in an individual similarly devoid of the Y chromosome [19]. It has been proposed that the degree of somatic mosaicism contributes to the extent of masculinization of freemartins, as observed in the current case. While typical freemartins with female genital hypoplasia are reported to have lower levels of male-derived chimerism [20], the extent and nature of chimerism in more masculinized freemartins remain insufficiently characterized. This case underscores how variations in tissue-specific cellular composition, in concert with differential expression and timing of sex-determining factors, may ultimately influence the trajectory of genital development.

## Conclusion

In conclusion, this case report describes a rare instance of XXX/XY chimerism in a Japanese black calf with complex urogenital malformations that differ from classical freemartinism or simple X-trisomy. While acknowledging the semi-quantitative nature of conventional PCR, the varied detection patterns of sex chromosome-specific loci across different tissues suggested a heterogeneous, organ-specific distribution of the chimeric cell lines, which likely contributed to the unique phenotype. This case illustrates the inherent plasticity of bovine sex determination and differentiation systems, stemming from cellular exchange during heterosexual twin pregnancies. These findings demonstrate that the developmental fate of the reproductive tract can be profoundly influenced by interactions between cell lines with discordant chromosomal constitutions, such as XX or XY [14, 19]. Anti-Müllerian hormone (AMH), which is controlled by the SRY gene, is expressed on the Y chromosome and produced by the testes, and AMH is a key factor for sex determination via the suppression of testicular differentiation and growth [21, 22]. Understanding the range of anomalies associated with such rare chimeric constitutions, particularly in twin cattle, is crucial for elucidating the mechanisms and flexibility of bovine sex determination [14]. This knowledge is vital not only for providing academic insight but also for improving the diagnosis and management of congenital reproductive disorders in economically important species.

## Data Availability

The datasets used and/or analysed during the current study are available from the corresponding author upon reasonable request.
